# Anthocyanins: Promising Natural Products with Diverse Pharmacological Activities

**DOI:** 10.3390/molecules26133807

**Published:** 2021-06-22

**Authors:** Jiaqi Liu, Hongbing Zhou, Li Song, Zhanjun Yang, Min Qiu, Jia Wang, Songli Shi

**Affiliations:** 1 Department of Pharmacy, Baotou Medical College, Baotou 014040, China; ljq15164771109@163.com (J.L.); zhouhongbing212@126.com (H.Z.); 102006095@btmc.edu.cn (M.Q.); 2 Institute of Bioactive Substance and Function of Mongolian Medicine and Chinese Materia Medica, Baotou Medical College, Baotou 014060, China; hhhtsongli@126.com (L.S.); yzj8330@163.com (Z.Y.)

**Keywords:** anthocyanin, natural products, systemic diseases, anticancer, anti-infection

## Abstract

Anthocyanins are natural products that give color to plants. As natural plant pigments, anthocyanins also have a series of health-promoting benefits. Many researchers have proved that anthocyanins have therapeutic effects on diseases, such as circulatory, nervous, endocrine, digestive, sensory, urinary and immune systems. Additionally, a large number of studies have reported that anthocyanins have an anticancer effect through a wide range of anti-inflammatory and antioxidant effects. The anti-disease impact and mechanism of anthocyanins are diverse, so they have high research value. This review summarizes the research progress of anthocyanins on the pharmacological agents of different diseases to provide references for subsequent research.

## 1. Introduction

Anthocyanins are natural water-soluble flavonoids. In Greek, anthocyanin means “blue flower”. As natural plant pigments, anthocyanins provide plants with various colors; for example, they give blue fruits and red petals their respective colors [1,2]. Studies have revealed that the color of anthocyanins is closely related to the pH value; they appear red under acidic conditions and turn blue or colorless as pH increases [3]. As secondary metabolites, anthocyanins are produced by plants under environmental stress conditions, including drought, cold temperature, and UV light, and therefore, play a major role in plant physiology [4]. Anthocyanins mainly exist as heterosides in nature. There are four commonly substituted monosides: arabinose, rhamnose, glucose and galactose. Over 600 anthocyanins have been extracted and isolated from plants. Specifically, malvidin (Mv), pelargonidin (Pg), delphinidin (Dp), petunidin (Pt), peonidin (Pn), and cyanidin (Cy) are widely distributed in plants [5]. The contents of these anthocyanidins in the edible parts of plants are Cy, 50%; Pg, 12%; Pn, 12%; Dp, 12%; Pt, 7%; and Mv, 7%. As important natural products, anthocyanins are important components of berry fruits. There are many foods rich in anthocyanins in the human diet, with blueberries having the highest anthocyanin content [6]. In addition, anthocyanins can be extracted from brightly colored crops such as strawberries, black currants, grapes, mulberries, black raspberries, cherries, purple rice, black beans, purple corn and purple sweet potatoes [7,8]. Therefore, anthocyanins are intense natural pigments.

**Figure 1:**
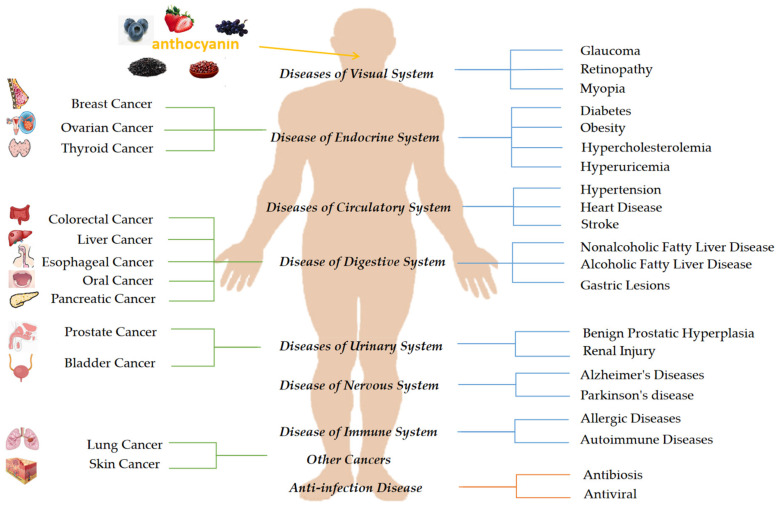
Summary of diseases prevented by anthocyanins.

The structure and characteristics of natural products have evolved due to changes in the natural climate over millions of years, and natural products can promote health by their protective action against disease. Natural products can often be used as alternatives for treating emerging conditions [9,10]. Due to the side effects of drugs and people’s positive attitudes towards natural foods, natural products are more accepted by people to prevent and treat diseases. Anthocyanins are widely found in fruits and vegetables and can be easily found in daily life. In the past 20 years, research has revealed that anthocyanins are nontoxic natural pigments that have antioxidant and anti-inflammatory effects. Anthocyanins also possess antimicrobial, antiviral, antiallergic, anticarcinogenic, anti-inflammatory, antimutagenic, and antiproliferative effects, and thus, may play an essential role in preventing various degenerative diseases. Research data have shown that the consumption of anthocyanin-rich foods may reduce the incidence of circulatory, nervous, endocrine, digestive, sensory, urinary, and immune system diseases and cancer. We have summarized the effects of anthocyanins from different plants on various conditions to provide a reference for anthocyanins as natural medicines for the treatment and prevention of diseases (Figure 1).

## 2. Effects Against Systemic Diseases

Dietary intake of anthocyanins has a spectrum of therapeutic effects against many systemic diseases, such as disease of the circulatory, nervous, endocrine, sensory, digestive, immune and urinary systems (Table 1). The primary mechanism of anthocyanins is inflammatory inhibition and a reduction in oxidative stress. The effects of anthocyanins against systemic diseases and the related mechanisms are discussed in the subsections below.

### 2.1. Diseases of the Circulatory System

#### 2.1.1. Hypertension

Anthocyanins and flavonoids may prevent hypertension. Anthocyanin-rich berries and red grapes/wine significantly reduce blood pressure [69], particularly in elderly individuals over 50 years old [70]. Observations on tens of thousands of women and men for 14 years indicated that people who consume more anthocyanins had a lower risk of hypertension [71]. An increased risk of cardiovascular disease is linked to endothelial dysfunction. Endothelium-derived nitric oxide (NO) deficiency is closely associated with hypertension [72]. Black currant extract (BCE) contains high anthocyanins concentrations and increases NO synthesis via endothelial nitric oxide synthase, which are critical regulators of cardiovascular disease [11]. Anthocyanin-enriched extracts of *Odontonema strictum* flowers can block the contraction of aortic rings because anthocyanin (400 μg/mL) inhibits the effects of CaCl_2_ and a thromboxane A_2_ analog agonist (U46619) in physiological salt solution [12]. Blood pressure was significantly reduced after consumption of 300 mL of anthocyanin-rich cherry in older adults [73]. A review of 66 experiments on the effect of anthocyanins from whole berries, berry juices, powders, purees and whole phenolic extracts on blood pressure showed that the dose, duration, content, and bioavailability of anthocyanins and individual differences in anthocyanin absorption and metabolism are significant factors conducive to the beneficial effect of anthocyanins on blood pressure [74].

#### 2.1.2. Heart Disease

It is generally believed that the cardioprotective effects of anthocyanins are based on their antioxidant properties. Oxidative stress can cause cardiomyocyte apoptosis and impair the function of these cells. Lingonberry anthocyanins can protect cardiac cells from apoptotic cell death induced by oxidative stress [13]. Diabetes causes cardiovascular complications, including myocardial infarction, ischemic heart disease, and cardiomyopathy [75]. Chen et al., studied the effect of anthocyanins extracted from purple rice on the hearts of streptozotocin (STZ)-induced type 1 diabetes mellitus (DM) rats. They found that anthocyanins inhibited the expression of TLR4/NFκB and molecular markers associated with cardiac hypertrophy and fibrosis [14]. Liu et al., found that in an STZ-induced DM rat model, oral administration of black rice anthocyanin significantly reduced cardiomyocyte apoptosis and significantly increased IGFIR/PI3K/protein kinase B (Akt) survival signaling, thus protecting the cardiac functions of DM rats [15]. The proposed mechanisms of the pharmacological cardioprotective effects of anthocyanins on mitochondria prevent apoptosis by reducing cytosolic cytochrome c expression and promotion of oxidative phosphorylation in ischemia-damaged mitochondria through maintenance of electron transfer between NADH dehydrogenase and cytochrome c [76]. In summary, anthocyanin extracts and pure individual anthocyanins from anthocyanin-containing plants have great potential as cardioprotective food ingredients or pharmacological compounds.

#### 2.1.3. Stroke

Stroke is a significant disease that causes death and disability in countries around the world. Redox imbalance leading to endothelial dysfunction is a major risk factor for stroke. Anthocyanins have the potential to limit and offset the effects of specific factors that are harmful to endothelial cells [77]. Anthocyanins from purple sweet potato relieved ischemic stroke by reducing the levels of apoptosis-inducing factors and enhancing brain-derived neurotrophic factor signaling, which is essential for stroke recovery, and other antioxidant mechanisms [16,78]. Based on the pathogenesis of cerebral ischemic injury and the anti-inflammatory and antioxidant effects of anthocyanins, it has been demonstrated that anthocyanins exert a protective effect against middle cerebral artery occlusion/reperfusion injury, and anthocyanins can inhibit the JNK-p53 signaling pathway and protect against stroke-induced neuronal damage [79]. Therefore, anthocyanins and their metabolites related to the vascular endothelium can prevent cardiovascular disease, including stroke.

### 2.2. Diseases of the Endocrine System

#### 2.2.1. Diabetes

DM is a serious chronic hereditary endocrine system disease characterized by high blood glucose concentrations [80]. Mulberry anthocyanin extract can alleviate pathological changes in diabetic mice by activating the PI3K/AKT pathway and reducing insulin resistance in HepG2 cells [17]. Black soybean seed coat extract (BSSCE), which contains cyanidin-3-glycoside and proanthocyanidins, improved insulin sensitivity and reduced blood sugar levels in type 2 DM mice. BSSCE may regulate GLUT4 and gluconeogenesis in skeletal muscle by activating AMPK [18]. BCE is rich in anthocyanins, including delphinidin 3-rutinin (D3R), and may help reduce DM medications and prevent diabetes. This is consistent with the fact that BCE stimulates glucagon-like peptide-1 (GLP-1) expression and induces insulin secretion to significantly improve glucose tolerance [19]. Differentiation of fat cells into smaller insulin-sensitive fat cells is also an important strategy for the treatment of diabetes. BSSCE and its active ingredient C3G reduced 3T3-L1 preadipocyte differentiation, activated skeletal muscle metabolism, and exerted antidiabetic effects in db/db mice [20]. Therefore, anthocyanin-rich extracts from plants have noticeable benefits for the treatment of diabetes.

#### 2.2.2. Obesity

Division and differentiation of preadipocytes increases the number of adipocytes and leads to obesity. Therefore, inhibiting the differentiation of adipocytes and adipogenesis is an effective way to fight obesity. Anthocyanins exert a strong anti-obesity effect through this mechanism. Han et al., reported that anthocyanins from *Vitis coignetiae* can effectively enhance the activation of AMPK and inhibit the expression of adipocyte-specific genes such as adipocyte fatty acid-binding protein, leptin, and fatty acid synthase [21]. Anthocyanins from *Prunus cerasus* can effectively reduce the expression of proinflammatory cytokines in adipocytes and improve antioxidant status in obesity [22]. Anthocyanins extracted from the fruit of *Aroina melanocarpa* suppressed visceral fat accumulation and hyperglycemia by inhibiting pancreatic lipase activity and intestinal lipid absorption [23]. Thus, anthocyanins from plants clearly have anti-obesity applications.

#### 2.2.3. Hypercholesterolemia

Hypercholesterolemia is characterized by metabolic disorders and elevated blood cholesterol concentrations. Anthocyanins extracted from black rice (*Oryza sativa*) can reduce cholesterol absorption via inhibition of pancreatic lipase activity, decrease cholesterol solubility in micelles and suppress cholesterol uptake in enterocytes [24]. Consistent intake of black raspberry (*Rubus occidentalis*) extract can reduce cecal trimethylamine and serum trimethylamine-N-oxide levels in rats, thus alleviating hypercholesterolemia and hepatic inflammation caused by excessive choline supplied by a high-fat diet [25]. Cranberry anthocyanin promotes the excretion of sterols with a neutral or acidic pH through the feces, which alters plasma lipoprotein profiles by reducing plasma total cholesterol (TC) levels, non-high-density lipoprotein cholesterol (HDL-C) levels, and the non-HDL-C/HDL-C ratio [26]. Concentrations of 0.5 and 1% blueberry anthocyanins reduce plasma TC concentration in a dose-dependent manner. This may be because blueberry anthocyanins increase sterol excretion and downregulate the gene expression of NPC1L1, Acat 2, MTP and ABCG 8 in the intestinal tract [27]. These findings provide important evidence for the use of anthocyanins as edible natural products for the prevention and treatment of hypercholesterolemia.

#### 2.2.4. Hyperuricemia

Hyperuricemia results from excessive uric acid production or inadequate renal excretion of it, and is characterized by high serum urate concentrations [81]. Anthocyanins from bilberry (*Vaccinium myrtillus*) and black currant (*Ribes nigrum*) can inhibit the activity of xanthine oxidase in the serum and liver, resulting in the inhibition of urate production. Moreover, urate reabsorption is decreased and urate excretion is increased through regulation of the levels of organic anion transporters [28]. Highly acylated anthocyanins from purple sweet potato can not only alleviate oxidative stress by regulating serum total superoxide dismutase (SOD) activity and MDA levels but also downregulate the protein expression of typical cytokines by mediating the NF-κB pathway [29]. Therefore, anthocyanin-rich foods have the potential to reduce the infiltration of inflammatory cells and alleviate kidney damage, which would alleviate hyperuricemia.

### 2.3. Diseases of the Digestive System

#### 2.3.1. Nonalcoholic Fatty Liver Disease

The causes of nonalcoholic fatty liver disease (NAFLD) are central obesity, type 2 DM, insulin resistance and other insulin resistance syndromes, and hyperlipidemia, not the ingestion of a large amount of alcohol [82]. Sweet cherry anthocyanins protect against hepatic steatosis not only through the peroxisomal proliferator-activated receptor signaling pathway and fatty acid metabolism but also through steroid and unsaturated fatty acid biosynthesis [30]. Anthocyanin extract of *Hibiscus sabdariffa* calyces protects hepatic tissue and alleviates the negative effect of thioacetamide on hepatocyte architecture through antioxidant and anti-inflammatory mechanisms [31]. Anthocyanin extracts from chokeberry, wild blueberry, strawberry, and blackberry can reduce the risk of NAFLD by exerting antioxidant and inhibitory effects against oleic acid (OA)-induced hepatic steatosis [32]. It has been demonstrated that bilberry anthocyanins can ameliorate Western diet-induced NAFLD by alleviating gut microbiome dysbiosis and dyslipidemia [33]. C3G, an anthocyanin primarily extracted from honeyberry (*Lonicera caerulea*), might resist hepatic steatosis by targeting AMPK-mediated fatty acid metabolism in the liver [34]. Franklin et al., demonstrated that grape leucoanthocyanidin can prevent NAFLD through its antioxidant properties [35]. In addition, cherry anthocyanins exert positive effects against OA-induced hepatic lipid accumulation by activating autophagy [36]. Therefore, anthocyanins from plant tissues have potential applications for the treatment of non-alcoholic diet-induced hepatic steatosis.

#### 2.3.2. Alcoholic Fatty Liver Disease

Alcoholic liver disease (ALD) is caused by alcohol abuse and is a significant liver disease worldwide [83]. Deacetylation of NF-κB and inactivation of the NLRP3 inflammasome may alleviate alcohol-induced hepatitis. Zhou et al., showed that physiologically available C3G could alleviate ALD through these mechanisms [37]. In addition, ALD is closely related to liver inflammation and excessive accumulation of lipids. Zuo et al., found that purified anthocyanins extracted from *Lonicera caerulea* can reduce inflammation and lipid accumulation by inhibiting proinflammatory cytokines and activating the AMPK pathway to prevent alcoholic hepatosteatosis [38]. Therefore, phytoanthocyanins can be developed as suitable products to protect against alcoholic hepatitis.

#### 2.3.3. Gastric Lesions

C3G may protect against gastric injury through its oxygen-free radical scavenging ability [39]. Orally administered *Vaccinium myrtillus* anthocyanoside significantly protects against gastric mucosal damage by decreasing lipid peroxide levels in a concentration-dependent manner [40]. Strawberry extract exerts a protective effect against ethanol-induced gastric injury, possibly by activating SOD and catalase and reducing lipid peroxidation reactions [41]. These studies suggest that anthocyanins from natural sources have a protective effect against gastric injury.

### 2.4. Diseases of the Urinary System

#### 2.4.1. Benign Prostatic Hyperplasia

Benign prostatic hyperplasia (BPH) is a chronic disease common in elderly male patients. BPH is associated with the proliferation of prostate cells such as smooth muscle cells, stromal cells and epithelial cells [84]. The prostate weight of rats with BPH induced by testosterone propionate is decreased after oral administration of polyanthocyanidins (PAs) extracted from grape skin. This effect is related to the reduced expression of androgen signaling pathway-related molecules and proliferation-related factors, prevention of the BPH-mediated increase in Bcl-2 expression, and increased expression of Bax [42]. Anthocyanin extract from bilberry (*Vaccinium myrtillus*) exerts an additive effect against stress-provoked BPH in mice when used in combination with the pollen of *Brassica napus* by decreasing lipid peroxidation levels, increasing oxygen radical absorbance capacity (ORAC) and glutathione (GSH) content, and elevating SOD and glutathione peroxidase (GPx) activity [43]. Anthocyanins derived from black soybeans can reduce prostate volume in BPH rats, and anthocyanins have clinical application value for BPH treatment [44]. Therefore, anthocyanins may be potential natural drugs for the prevention or treatment of BPH.

#### 2.4.2. Renal Injury

Anthocyanins from the berries of *Aronia melanocarpa* can alleviate acute renal failure by reducing oxidative stress and exerting cytoprotective effects [45]. Anthocyanins from bilberries enhance antioxidant activity by reducing the consumption and modification of antioxidant enzymes and thus, reduce the degree of damage to the distal and proximal tubules of the renal cortex [46]. Anthocyanins from *Glycine max* ameliorate diabetic nephropathy by activating the AMPK pathway and thereby inhibiting apoptosis and oxidative stress [47]. These studies suggest that the protective effects of naturally derived anthocyanins on the kidneys are primarily related to their antioxidant and anti-inflammatory effects.

### 2.5. Eye Diseases

#### 2.5.1. Glaucoma

Glaucoma occurs most frequently in elderly individuals and is one of the most common causes of irreversible blindness worldwide. Normal-tension glaucoma (NTG), which causes nerve damage when intraocular pressure (IOP) is normal, is a rarer type of glaucoma [85]. Oral administration of black currant anthocyanins (BCACs) may decrease IOP in healthy subjects and patients with NTG [48]. It has been suggested that *Ginkgo biloba* extract and anthocyanins extracted from *Vaccinium myrtillus* may be effective for improving visual function in patients with NTG by exerting effects on blood circulation and antioxidant effects [49]. Long-term oral administration of BCACs can inhibit the deterioration of the visual field in open-angle glaucoma (OAG) patients and alleviate glaucoma, possibly by acting on ETB receptors and thus, normalizing patients’ serum endothelin-1 concentrations and improving ocular blood flow [50]. Therefore, regular consumption of foods containing anthocyanins is an alternative to prevent or deaccelerate glaucoma development.

#### 2.5.2. Retinopathy

Oxidative stress and inflammation play an essential role in the development of retinopathy. The antioxidant and anti-inflammatory activities of anthocyanins can alleviate retinopathy and vision loss [86]. Anthocyanin-rich bilberry extract inhibits STAT3 activation, which reduces inflammation-related rhodopsin expression and reduces intracellular reactive oxygen species (ROS) levels, preventing photoreceptor cell damage and protecting visual function during retinal inflammation [87]. Anthocyanins from blueberries, as well as the principal constituent, Mv, and its glycosides, can protect human retinal capillary cells against high glucose-induced injury [51]. Anthocyanins from bilberries (*V. myrtillus*) protect retinal function and histological integrity by increasing antioxidant defense mechanisms, inhibiting lipid peroxidation and proinflammatory cytokine expression, and inhibiting retinal cell apoptosis [52]. Anthocyanins from black soybean seeds can prevent the damage to the retinal nerve caused by N-methyl-N-nitrosourea and can be used as therapeutic agents for the prevention and treatment of retinal degeneration [53]. Thereby, anthocyanins from plant tissues have the potential to prevent the progression of retinopathy.

#### 2.5.3. Myopia

Myopia is the most common disease of the eye and is a serious public health problem, especially in developing countries. Continuous spasms due to excessive contraction of ciliary muscle (CM) during reading and working at a close range and weakening of the spasmodic refraction of the lens are factors contributing to the rise in the incidence of myopia. The key mechanism underlying the treatment effect of anthocyanins against myopia is relaxation of the CM [88]. Anthocyanins from *Ribes nigrum* relax ciliary smooth muscle and relieve and prevent myopia [89]. This is based on the fact that the main component of D3R can inhibit CM contraction induced by endothelin-1 [54]. In summary, anthocyanins can improve vision health and treat and prevent eye diseases such as myopia, glaucoma, and retinopathy.

### 2.6. Diseases of the Nervous System

#### 2.6.1. Alzheimer’s Disease

Anthocyanins exert a protective effect on nerve tissue by crossing the blood–brain barrier [90]. Altered amyloid precursor protein (APP) processing potentiates the aggregation of glycation products, and amyloid-β (Aβ) toxicity is a key pathogenic feature of Alzheimer’s disease (AD) [91,92]. Anthocyanins from cranberries, black raspberries, blackberries, strawberries, red raspberries, and blueberries have free radical-scavenging, anti-Aβ aggregation, and anti-glycation effects and potential neuroprotective effects on microglia, which suggests that it may exert neuroprotective effects against AD [55]. Mulberry (*Morus atropurpurea*) anthocyanins can protect against aging-induced oxidative damage and cognitive deficits caused by antioxidant effects and has the potential to inhibit Aβ [56]. Vepsäläinen et al., suggested that long-term supplementation of bilberry or black currant to transgenic AD mice had beneficial effects on APP and Aβ metabolism [57]. The underlying antioxidant and neuroprotective mechanism of natural dietary Korean black bean has been demonstrated to be because anthocyanins regulate the PI3K/Akt/GSK3 pathway, reducing amyloid beta oligomer (AβO)-induced oxidative stress and preventing neurodegeneration via a PI3K/Akt/Nrf2-dependent pathway [58]. Katherine et al., found that giving anthocyanin-rich cherry juice to older adults with mild to moderate dementia for 12 weeks significantly improves cognition and short- and long-term memory [93]. Riham et al., tested the in vitro antioxidant potential anthocyanins extracted from two Hibiscus varieties (white and red calyces). They found that these anthocyanins benefit AD by exerting anti-amyloidogenic, anti-acetylcholinesterase, antioxidant, and anti-inflammatory activities [59]. Purple sweet potato anthocyanins can protect PC-12 cells and play a role in treating AD by inhibiting mitochondrial dysfunction, intracellular calcium flow, and eventually, cell apoptosis to resist the development of AD [60]. Therefore, supplementation of anthocyanins can prevent AD.

#### 2.6.2. Parkinson’s Disease

Parkinson’s disease (PD) and AD are both neurodegenerative diseases. PD results from the progressive loss of dopaminergic (DA) neurons in the substantia nigra. In addition, IGF-1 can inhibit DA neurotoxicity and protect nerves and thus, may play a role in PD treatment [94]. Anthocyanins prevent the deficits in working memory induced by Aβ or a long-term grain monodiet. Supplementation of BCACs increases cyclic glycine–proline concentration, which exerts neuroprotective effects by improving IGF-1 function [95]. Wheat rich in anthocyanins is a source of beneficial nutrients in the early stage of this neurodegenerative disease. It has been found that a diet consisting of wheat rich in anthocyanins reduces the accumulation of alpha-synuclein and regulates the microglial response in the brains of PD transgenic mice [61]. Khan et al., showed that anthocyanins boost hippocampus-dependent memory function, slowing inflammation-induced neurodegeneration in the brain to protect against PD via JNK/Akt/GSK3β signaling in lipopolysaccharide-treated adult mice [62]. Anthocyanin-rich *Morus nigra* fruit juice can significantly improve 1-methyl-4-phenyl-1,2,3,6-tetrahydropyridine (MPTP)- and levodopa-induced dyskinesia in PD individuals [63]. In addition, anthocyanin- and proanthocyanidin-rich extracts from Chinese mulberries, hibiscus, blueberries, black currants, and grape seeds may alleviate neurodegeneration in PD by enhancing mitochondrial function [64]. Therefore, a diet rich in anthocyanins counteract neurodegenerative diseases and can be used as a dietary supplement for such diseases.

### 2.7. Diseases of the Immune System

#### 2.7.1. Allergic Diseases

Allergic diseases are common chronic diseases that involve hypersensitivity of the immune system. Anthocyanins have the potential to treat allergic diseases. Deok et al., found that anthocyanin pigment from *Schisandra chinensis* ameliorates allergic inflammation by suppressing inflammatory cytokine expression in HMC-1 cells [65]. Oral administration of C3G from the black rice husk (*Oryza sativa*) has a significant alleviating effect on allergic airway inflammation by reducing the production of Th2 cytokines, the IL-4Rα-STAT6 signaling pathway and eosinophilic infiltration [66]. Oral supplementation with BCACs can effectively slow airway inflammation in mice with acute allergic lung inflammation [67]. Therefore, anthocyanins have the potential to alleviate allergies.

#### 2.7.2. Autoimmune Diseases

Rheumatoid arthritis (RA) is an autoimmune disease caused by chronic inflammation of the synovium of the joints, leading to the destruction of bone and cartilage. The balance between inflammatory T helper 17 (Th17) and regulatory T cells has emerged as a significant factor in autoimmunity [96]. Anthocyanins extracted from black soybean seed coats can reduce the Th17 cell number in collagen-induced arthritis model mice and effectively inhibit the expression of proinflammatory cytokines and oxidative stress, thus mitigating autoimmune arthritis [68]. As an anthocyanin, delphinoside chloride can increase the secretion of TGF-β from Tregs, enhance T cells’ regulatory function, and treat excessive immune reactions, such as allograft rejection [97].

## 3. Anticancer Effects

Avoiding contact with carcinogens and consuming natural foods containing anticancer activities are ways to reduce the risk of cancer. Natural products and molecules related to their synthesis could effectively improve the body’s resistance to cancer [98]. Anthocyanins are natural polyphenols that are widely found in fruits and vegetables and have anticancer chemopreventive effects. There has been an increase in interest in anthocyanins, owing to their antioxidant and antiproliferative properties. Numerous studies have shown that anthocyanins have a very wide range of anticancer properties, and can inhibit the growth of many kinds of cancer cells by exerting cytotoxic effects, and inducing DNA damage to cause cell cycle arrest (Table 2). The anticancer effects and mechanisms of anthocyanins in different cancer types are discussed in the following sections.

### 3.1. Tumors of the Digestive System

#### 3.1.1. Colorectal Cancer

Colorectal cancer is strongly influenced by diet. Thus, dietary treatments are suitable for this disease. As important natural products, anthocyanins exert therapeutic and preventive effects against colorectal cancer by modulating the gut microbiota and regulating inflammation. Black raspberry anthocyanins might act as chemopreventive agents in colorectal cancer by promoting the growth of protective bacteria, and the regulation of the composition of the gut microbiota causes demethylation of the secreted frizzled-related protein gene promoter [99]. He et al., suggested that black raspberry anthocyanins exert their chemopreventive effect against colorectal cancer by downregulating the expression of β-catenin and its downstream target genes [100]. Anthocyanin-rich grape and strawberry extracts act as anticarcinogenic agents by exerting apoptotic effects in HT-29 colon cancer cells [101]. Anthocyanins extracted from the fruit of *Vitis coignetiae Pulliat* suppress the invasive ability of human colon cancer cells by inhibiting nuclear factor κB [102]. In addition, anthocyanins extracted from purple potato can reduce the incidence of colon cancer by enhancing mitochondria-mediated apoptosis in vivo through inhibition of the Wnt/β-catenin signaling pathway, thereby reducing the number of colon cancer stem cells [103]. Anthocyanin-rich purple-shoot tea extract (PET) is a potential new chemoprophylactic agent for colorectal cancer. Hsu et al., reported that PET could inhibit the proliferation of two types of human colorectal carcinoma cells (COLO 320DM and HT-29 cells) through a signaling pathway in a dose-dependent manner [104]. Thus, anthocyanins from plants have the potential to prevent and treat colon cancer.

#### 3.1.2. Liver Cancer

Anthocyanins extracted from purple rice bran can inhibit the expression of the inflammatory enzyme iNOS and the proinflammatory cytokines TNF-α and NF-κB, and ultimately reduce preneoplastic cell proliferation in mice with diethylnitrosamine (DEN)-induced early hepatocarcinogenesis, thus protecting against DEN-induced hepatocarcinogenesis [105]. Liao et al., found that 150 mg·kg^−1^ anthocyanins extracted from mulberries reduce the incidence of liver cancer induced by N-nitrosodiethylamine by half. The protective effect of anthocyanin extract against liver cancer is related to a decrease in the expression of the inflammatory mediator COX-2, which, through the NF-κB pathway, induces the expression of antioxidant enzymes and reduces lipid peroxidation [106]. As nutritional supplements, anthocyanins from blueberries can suppress the development of hepatocellular carcinoma by inhibiting invasion, apoptosis, migration, and proliferation-related pathways [107].

#### 3.1.3. Esophageal Cancer

Esophageal cancer is the sixth most common cancer in the world [124]. Anthocyanin-enriched fraction isolated from black raspberries can effectively alleviate N-nitrosomethylbenzylamine (NMBA)-induced esophageal tumorigenesis by inhibiting the expression of genes associated with inflammation in the esophagus via a reduction in the expression of biomarkers (COX-2, iNOS, p-NF-kB, and sEH) and cytokines (PTX3) [108]. Another mechanism by which black raspberry anthocyanins inhibit esophageal tumorigenesis is by altering cytokine expression and the trafficking of innate immune cells into tumor tissues [109].

#### 3.1.4. Pancreatic Cancer

Pancreatic cancer is an aggressive type of cancer characterized by metastasis, which involves cell adhesion, invasion, migration and the expression and secretion of several extracellular matrix-degrading proteolytic proteases [125]. Kuntz et al., reported that anthocyanins and their metabolites isolated from the plasma of healthy subjects who ate anthocyanin-rich fruits reduce pancreatic cancer cell migration in vitro, as determined by cell phenotypes [110].

#### 3.1.5. Oral Cancer

Anthocyanins from a species of black rice can suppress the in vitro migration and invasion of human oral cancer CAL 27 by reducing MMP-2, MMP-9, and NF-kB p65 expression through the suppression of the PI3K/Akt pathway and inhibition of NF-kB expression [111]. The blueberry anthocyanins can inhibit the proliferation of oral cancer KB cells in a dose-dependent manner by inducing G_2_/M cell cycle arrest and apoptosis, and downregulating the methylation of p53 [112]. Yue et al., found that anthocyanins promote the death of oral squamous cell carcinoma cells by activating pyroptosis [126].

### 3.2. Tumors of the Endocrine System

#### 3.2.1. Breast Cancer

Anthocyanin extract is a potential adjuvant therapy for breast cancer. Anthocyanins from grape skin can markedly increase intracellular ROS levels and apoptosis of MCF-7 breast cancer cells and arrest cells in the G2/M phase [113]. In addition, Alba strawberry anthocyanin extract can induce apoptosis and death of breast cancer cells by exerting antioxidant activity and downregulating AMPK expression, which plays a role in resisting breast cancer [114]. *Eugenia jambolana* fruit extract, which contains 3.5% anthocyanins, exhibits proapoptotic effects against breast cancer cells but not against normal breast cells [115]. The anthocyanin cya-3-O-sam, extracted from the fruit of *Acanthopanax sessiliflorus*, inhibits metastasis of breast cancer cells by suppressing neovascularization and the gelatinolytic activity of MMP-9 [116]. Additionally, black rice anthocyanins inhibit the metastasis of breast cancer cells by targeting the Ras/Raf/MAPK pathway [117].

#### 3.2.2. Ovarian Cancer

It is difficult to treat ovarian cancer due to chemotherapy drug resistance, and reducing drug resistance has become a key focus of cancer treatment [127]. Anthocyanins can reduce the effective dose of cisplatin required for the treatment of ovarian cancer and reduce drug resistance. Anthocyanidins isolated from bilberries can effectively treat ovarian cancer by reducing the resistance of ovarian cancer cell lines to overexpression of p-glycoprotein [118]. Delphinidin suppressed brain-derived neurotrophic factor-induced ovarian cancer migration and invasion through decreasing Akt activation [128].

#### 3.2.3. Thyroid Cancer

Thyroid cancer is a common malignancy of the endocrine system. Activation of the Akt/mammalian target of the rapamycin (mTOR) pathway is critical during nutrient-induced autophagy and is closely related to thyroid cancer cells. Long et al., suggested that mulberry anthocyanins exert antitumor effects against thyroid cancer cells by suppressing Akt, mTOR, and ribosomal protein S6, expressing and inducing SW1736 and HTh-7 cell death in a manner that is partially dependent on autophagy [119].

### 3.3. Prostate and Bladder Cancer

A natural nontoxic anthocyanin diet can inhibit the growth and development of prostate cancer. It has been found that grape seed extract proanthocyanidins significantly reduce constitutive and Jagged1 (Notch1 ligand)-induced activation of the Notch1 pathway to target prostate cancer growth and tumor recurrence [120]. Rice bran anthocyanins, cyanidin-3-glucoside, inhibit the progression of PC_3_ prostate cancer cells due to the inhibition of epithelial mesenchymal transition through Smad signaling pathway(s) mediating Snail/E-cadherin expression [121].

Purple sweet potato anthocyanins can reduce the viability of bladder cancer cells in a dose-dependent manner. Li et al., demonstrated that inhibition of the PI3K/Akt signaling pathway can aggravate loss of the mitochondrial membrane potential, promote cell apoptosis, and induce cell cycle arrest, which are the key mechanisms of the anticancer effect of anthocyanins from purple sweet potato [122]. The effect of purple sweet potato anthocyanins on the apoptosis of bladder cancer BIU87 cells is dose-dependent [129].

### 3.4. Other Cancers

The combination of Cy, Mv, Pn, Pt and Dp at suboptimal doses can synergically inhibit the proliferation and metastasis potential of non-small cell lung cancer cells by regulating the WNT, Notch and NFkB signaling pathways and enhancing cell cycle arrest and apoptosis [123]. The primary mechanism of skin cancer is oxidative stress. Dp is one of the most effective and widely distributed anthocyanins in plants. Dp can activate the NRF2-ARE pathway, which is associated with antioxidant activity. Anthocyanins may be used as natural supplements for skin cancer [130].

## 4. Effects Against Infectious Disease

The antimicrobial and antiviral effects of anthocyanin and the related mechanisms of action are reviewed below (Table 3).

### 4.1. Antimicrobial Effects

Anthocyanins can be used as alternative antimicrobial agents. Blueberry anthocyanins interfere with *Staphylococcus aureus* and *Escherichia coli* growth, inhibit the formation of biofilms, and hinder bacterial adhesion without reducing bacterial growth, which is the mechanism by which anthocyanins prevent the development of drug resistance and infection [131]. Anthocyanins extracted from *Aronia niflora* and an antibiotic for urinary tract infections synergistically and significantly inhibited the formation of monoculture biofilms in 11 tested strains [132]. Anthocyanidins from black mulberries (*M. nigra*) exert strong analgesic and antimicrobial effects against *S. aureus*, *Pseudomonas aeruginosa* and *E. coli* by inhibiting the expression of proinflammatory cytokine-, iNOS- and NF-κB pathway-related proteins [133]. Anthocyanins from *Syzygium cumini* can be used as novel agents for sensing regulatory phenotypes based on a reduction in violacein production, biofilm formation and EPS production of *Klebsiella pneumoniae* in a concentration-dependent manner [134]. Anthocyanin extracts from bilberry (*V. myrtillus*) and blueberry (*Vaccinium corymbosum*) have antimicrobial properties involving antioxidant activity [135]. Anthocyanin extract from black flour can inhibit the growth of *Candida albicans*, *P. aeruginosa*, *E. coli*, and *S. aureus* [136]. Aichinger et al., studied the effect of altertoxin II on the cytotoxic effects of Dp on HT-29 colon cancer cells and showed that the concentration of mycotoxin altertoxin II is reduced in the presence of anthocyanins and that anthocyanins can protect the gut tract from genotoxicity induced by altertoxin II [141]. Therefore, the natural antimicrobial properties of anthocyanins expand their application prospects in the pharmaceutical and food industries.

### 4.2. Antiviral Effects

The chemical structure of anthocyanins plays a crucial role in their ability to inhibit viral activity. Hayashi et al., found that Pg-type anthocyanins isolated from red-fleshed potato can inactivate influenza viruses A and B [137]. Kannan et al., demonstrated that viruses are susceptible to natural cyanidin-3-sabubiocide and that cyanidin-3-sabubiocide can treat H1N1 subtype influenza virus [142]. Anthocyanins from elderberry fruit have potential as antiviral drugs for SARS CoV-2 by preventing reproduction via budding from the host cell of the virus [138]. Some anthocyanin-related substances in small red beans (*Vigna angularis*) can affect the early stage of rabies virus infection and the infectivity of the rabies virus [139]. In addition, oligomeric proanthocyanidins from *Crataegus sinaica* have apparent inhibitory effects on herpes simplex virus type 1 (HSV-1) [140].

## 5. Conclusions

Anthocyanins are natural flavonoids that can alleviate a variety of systemic diseases and cancers and have antiviral and bacterial properties. Anthocyanins have pharmacological potential for diseases of the circulatory, endocrine, digestive, urinary, sensory, nervous and immune systems. Studies have shown that anthocyanins can alleviate circulatory system diseases, mainly by increasing NO synthesis and inhibiting the jnk-p53 signaling pathway and antioxidative stress. To treat endocrine system diseases, anthocyanins can ameliorate insulin sensitivity, activate the PI3K/Akt pathway and reduce insulin resistance in HepG2 cells. Adipocyte differentiation is inhibited, the AMPK signaling pathway is activated, urate reabsorption is decreased and urate excretion is increased. Anthocyanins can also be used to treat diseases of the digestive and urinary system by acting as anti-inflammatory and antioxidant agents to activate the AMPK pathway and promote apoptosis by exerting anti-lipid peroxidation effects. Anthocyanins can alleviate visual diseases, mainly by affecting blood circulation and exerting antioxidative effects and can combat immune system diseases by reducing eosinophil infiltration and Th2 and Th17 cell development.

Accumulating evidence suggests that anthocyanins have therapeutic effects against various cancers, inhibiting the growth of a variety of tumor cells by exerting cytotoxic effects, causing DNA damage-induced cell cycle arrest and suppressing PI3K/Akt signaling. In addition, anthocyanins can regulate the intestinal symbiotic flora, exert anti-inflammatory and antiproliferative effects to prevent colorectal cancer and inhibit proliferation-, apoptosis-, migration- and invasion-related pathways and other related pathways to inhibit liver cancer. Anthocyanins can exert anti-inflammatory effects and alter the expression of cytokines and the number of innate immune cells in tumor tissues to prevent esophageal cancer. By reducing NF-κB, MMP-2 and MMP-9 expression, anthocyanins can inhibit the metastasis of pancreatic and oral cancer. Anthocyanins can also induce apoptosis and death of breast cancer cells by arresting cells in the G2/M phase and downregulating AMPK expression.

Anthocyanins also exert important antibacterial and antiviral effects. Several studies have reported that *K. pneumonia*, *P. aeruginosa, S. aureus*, *E. coli*, IVA and IVB, H1N1, SARS CoV-2, rabies virus, and HSV-1 are sensitive to anthocyanins.

Currently, people’s living standards are increasingly improving. People can easily consume diets rich in anthocyanins derived from many different fruits and plants. Many studies have found that strawberries, black currants, grapes, mulberries, black raspberries, cherries, purple rice, black beans, purple corn and purple sweet potatoes are rich in anthocyanins. Anthocyanins have a wide range of pharmacological effects and high potential for therapeutic development. More attention should be given to anthocyanins’ therapeutic and preventive mechanisms in different diseases to promote the development and utilization of anthocyanins to fight more diseases.

## Figures and Tables

**Table 1:** Mechanisms of anthocyanins from different plants on different systemic diseases.

Diseases	Plant Origin	Anthocyanin Types	Mechanism	Reference
High Blood Pressure	*Ribes nigrum*	C3G, C3R, D3G, D3R	↑eNOS mRNA levels ↑NO synthesis	[11]
	*Odontonema strictum*		↓CaCl2 and U46619 effect	[12]
Diabetic Heart Disease	*Vaccinium vitis-idaea*	C3G, C-3-Ara, C-3-Gal	↑Antioxidant	[13]
	*Oryza sativa*	C3G	↓TLR4/NFκB ↓active hypertrophy ↓Fibrosis associated molecular marker	[14]
	*Oryza sativa*	C3G	↑Survival signals ↓Apoptosis and the associated proapoptotic proteins	[15]
Stroke	Purple potatoes		↓AIF ↓Apoptosis ↑BDNF	[16]
Diabetes	*Morus alba*	C-3-Ara, C-3-Gal, C3G	↑PI3K/AKT	[17]
	*Glycine max*	C3G	↑AMPK ↑Modulates GLUT4 ↓Gluconeogenesis	[18]
	*Ribes nigrum*	D3R	↑GLP- 1	[19]
	*Glycine max*	C3G	↑Skeletal muscle metabolism ↑3T3-L1 preadipocytes differentiation	[20]
Obesity	*Vitis coignetiae*		↓Adipocyte differentiation ↓Adipogenesis	[21]
	*Prunus cerasus*	C3G, C3R, C3GR	↓Leptin and IL-6 ↑Antioxidant ↑SOD	[22]
	*Aronia melanocarpa*	C-3-Gal, C-3-Ara, C3G	↓Pancreatic lipase activity ↓Intestinal lipid absorption.	[23]
Hypercholesterolemia	*Oryza sativa*	C3G, Pn-3-G	↓Pancreatic lipase activity ↓Cholesterol solubility in micelles ↓Cholesterol uptake in enterocytes	[24]
	*Rubus occidentalis*	C3G, C3R, C3XR	↓Cecal TMA ↓Serum TMAO	[25]
	Cranberry	C-3-Gal, C-3-Ara, Pn-3-Gal, Pn-3-Ara	↑Excretion of fecal neutral and acidic sterols	[26]
	*Vaccinium ashei*	C3G, P3G	↑Excretion of sterols ↓NPC1L1, ACAT, MTP, ABCG 8	[27]
Hyperuricemia	*Vaccinium myrtillus/* *Ribes nigrum*	C3G, D3G	↓Urate production ↑Uric acid excretion	[28]
	*Ipomoea batatas*	Acylated Anthocyanins	↓Oxidative stress ↓Infiltration of inflammatory cells	[29]
Nonalcoholic Fatty Liver Disease	*Prunus auiun*	C3GR, C3R, Pg-3-R	Changed PPAR signaling pathway	[30]
	*Hibiscus sabdariffa*	D-3-Gal, D-3-Sam, D-3-Gen, D-3-Neo, C-3-Sam	↓Antioxidant ↓anti-inflammatory ↑SOD ↑INF-c	[31]
	*Rubus sp*	C3G	↑Antioxidants ↓TG accumulation in HepG2 cells	[32]
	*Vaccinium spp*	Mv-3-G, D3G, Pt-3-G, D-3-Ara, D-3-Gal, C3G		
	*Fragaria ananassa*	Pg-3-G		
	*Aronia melanocarpa*	C-3-Gal, C-3-Ara, C3G, C3X		
	*Vaccinium myrtillus*	Dp, cy, pt, Pn, Mv	↓Dyslipidemia ↓Gut microbiome dysbiosis	[33]
	*Lonicera caerulea*	C3G	↓Hepatic lipid metabolic gene expression ↑AMPK ↑ACC	[34]
	*Vitis vinifera*	leucoanthocyanidin	↑Antioxidant	[35]
	*Fragaria x ananassa*	C3G, C3R, Pt-3-G	↑Autophagy	[36]
Alcoholic Fatty Liver Disease	*Oryza sativa*	C3G	↓NLRP3 ↓NF-κB,	[37]
	*Lonicera caerulea*	C3G	↑AMPK ↓Lipid accumulation ↓F4/80 ↓IL-1β	[38]
Gastric Lesions	Purple corn husks	C3G	↑Glutathione ↑Radical scavenging enzymes	[39]
	*Vaccinium myrtillus*	Dp, Cy, Mv	↑Antiperoxidative	[40]
	*Fragaria x ananassa*	Pg-3-G, Pg-3-MG, Pg-3-R	↑Antioxidant enzymes	[41]
Benign Prostatic Hyperplasia	*Vitis vinifera*	PA	↓AR, 5AR2, SRC1, PSA, PCNA ↓DHT ↓PCNA ↓Cyclin D1 ↑Bcl-2, Bax	[42]
	*Vaccinium myrtillus*	D-3-Gal, D3G, D-3-Ara, C3G, Mv-3-G, Pt-3-G	↓Lipid peroxidation level ↑ORAC, GSH ↑SOD, GPx	[43]
	*Glycine max*	D3G, C3G, Pt-3-G	↑Apoptosis	[44]
Renal Injury	*Aronia melanocarpa*	C-3-Ara, C3G, C-3-Gal	↓Proinflammatory cytokines ↓Oxidative stress ↓Lipid peroxidation ↓Apoptosis	[45]
	*Vaccinium myrtillus*	D-3-Gal	↑Antioxidant ↑Anti-inflammatory	[46]
	*Glycine max*	D3G, C3G, Pt-3-G	↑AMPK	[47]
Glaucoma	*Ribes nigrum*	D3G, D3R, C3G, C3R	↑ETB receptor ↓Ocular blood vasodilation	[48]
	*Vaccinium myrtillus*		↑Blood circulation, Antioxidant	[49]
	*Ribes nigrum*		Modulate ET-1 ↑ocular blood flow	[50]
Retinopathy	*Vaccinium ashei*	Mv-3-G	↑Antioxidant, anti-inflammatory	[51]
	*Vaccinium myrtillus*	Dp, Mv, Pt, Cy, Pn	↑Antioxidant ↓Lipid peroxidation ↓Proinflammatory cytokines ↓Retinal cells apoptosis.	[52]
	*Glycine max*	C3G	↓GFAP ↑Anti-inflammatory	[53]
Myopia	*Ribes nigrum*	D3R, D3G, C3R, C3G	↑NO ↓RLC	[54]
Alzheimer’s Syndrome	*Rubus sp*	C3G, C-3-Ara, C3X, C3MG, C3DG	↓Free radical ↑Anti-Aβ aggregation, Anti-glycation	[55]
	*Rubus occidentalis*	C-3-Sam, C3G, C3XR, C3R		
	*Vaccinium angustifolium*	C-3-Gal, Pt-3-Gal, Pt-3-G, Pn-3-Gal, Mv-3-G		
	*Vaccinium macrocarpon*	C3G, C-3-Ara, Pn-3-Gal, Pn-3-Ara		
	*Rubus idaeus*	C3G, C-3-Ara, D-3-Ara		
	*Fragaria ananassa*	C3G, Pg-3-G, Pg-3-R		
	*Morus atropurpurea*		↓Oxidative stress ↓JNK, p38 ↑ERK ↓Accumulation of Aβ	[56]
	*Ribes nigrum*	D3G, D3R, C3G, C3R	↓Aβ40 ↓Aβ42	[57]
	*Vaccinium myrtillus*			
	*Glycine max*	C3G, D3G, Pt-3-G	↑Antioxidant Regulated the PI3K/Akt/GSK3 pathway	[58]
	*Hibiscus sabdariffa*	C3G	↓Inflammatory ↓Acetylcholinesterase ↓Amyloidogenic ↑Antioxidant	[59]
	*Ipomoea batatas*	Cy, Pn	↓Oxidative damage ↓Intracellular calcium ↓Influx, Mitochondria dysfunction ↓Cell apoptosis	[60]
Parkinson’s disease	wheat grain	C3G	↓Alpha-synuclein Modulated microglial response	[61]
	*Glycine max*	C3G, D3G, Pt-3-G	↓P-NF-kB, TNF-α, IL-1β ↑P-Akt, p-GSK3β, Bcl-2	[62]
	*Morus nigra*	C3G	Antioxidant	[63]
	*Vaccinium corymbosum*	Cy, Dp, Mv, Pn, Pt	↑Neuroprotective activity; Disrupting toxicant entry into the cells	[64]
	*Ribes nigrum*	D3G, C3G	↓Microglial activation; Amelioration of mitochondrial dysfunction	
Allergic Diseases	*Schisandra chinensis*	C3R	↓inflammatory cytokines	[65]
	*Oryza sativa*	C3G	↓IL-4, IL-5, IL-13 ↓IL-4Rα-STAT6 ↓Inflammatory cell infiltration and mucus hyper-production	[66]
	*Ribes nigrum*	Dp, Cy	↓Inflammation ↓Eosinophilia	[67]
Autoimmune Diseases	*Glycine max*	C3G, D3G, Pt-3-G	↓NF-κB ↓Osteoclastogenesis ↓Oxidative stress	[68]

**Table 2:** Mechanisms of anthocyanins from different plants on different cancers.

Diseases	Plant Origin	Main Anthocyanins	Mechanism	Reference
Colorectal Cancer	*Rubus occidentalis*	C3G, C3XR, C3R	↑Probiotics ↓Inflammation ↓Pathogenic bacteria	[99]
	*Rubus occidentalis*	C3G, C3XR, 3CR	↑MiR-24-1-5p ↓β-catenin	[100]
	*Vitis vinifera*	Mv	↓HT-29 colon cancer cells	[101]
	*Fragaria ananassa*	Pg-3-G		
	*Vitis coignetiae Pulliat*	D-3-5-D, Pt-3-5-D, D3G, Mv-3-5-D, Pn-3-5-D, C3G, Pt-3-G, Pn-3-G, Mv-3-G	↓NF-jB ↓MMP-2 ↓MMP-9	[102]
	*Solanum tuberosum*	Mv-3-RG Pt-3-RG	↓Wnt/β-catenin ↑Mitochondria-mediated apoptosis	[103]
	purple-shoot tea	Dp, Cy, Mv, Pn	↑Caspase 3 ↑Bax/Bcl-2	[104]
Liver Cancer	*Oryza sativa*	C3G, Pn-3-G	↓TNF-α, iNOS, NF-κB ↓Cell proliferation	[105]
	*Morus alba*	C3G, C3R	↓Lipid peroxidation ↓COX-2 ↑Nrf2-mediated antioxidant enzymes	[106]
	*Vaccinium angustifolium*	Mv-3-Gal	↓Proliferation, apoptosis, migration, Invasion-related pathways	[107]
Esophageal Cancer	*Rubus occidentalis*	C3G, C3R, C3XR	↓Genes associated with inflammation	[108]
			Altering cytokine expression and innate immune cell trafficking into tumor tissues.	[109]
Pancreatic Cancer	*Vitis vinifera*		↓Pancreatic cancer cell migration in dependency of the phenotype of cells	[110]
	*Vaccinium myrtillus*			
Oral Cancer	*Oryza sativa*	C3G	↓MMP-2, MMP-9 ↓NF-Kb ↓NFkB p65↓ PI3K/Akt	[111]
	*Vaccinium uliginosum*	C3G	↓methylation of p53 ↑caspase-9 ↑cytochrome c	[112]
Breast Cancer	*Vitis vinifera*	D-3-5-D, C3R	↑Intracellular reactive oxygen ↑Apoptosis ↓MCF-7 cells in the G2/M phases	[113]
	*Fragaria x ananassa*	C3G, Pg-3-G	↓AMPK ↑Apoptosis ↑Oxidative stress	[114]
	*Eugenia jambolana*	Dp, Cy, Pt, Pn, Mv		[115]
	*Acanthopanax sessiliflorus*	C-3-Sam	↓Metastasis processes, regulation of matrix metalloproteinase9 activity	[116]
	*Oryza sativa*		↓Metastasis in breast cancer cells by targeting the RAS/RAF/MAPK pathway	[117]
Ovarian Cancer	*Vaccinium myrtillus*	Dp, Cy, Mv, Pe, Pt	↓p-glycoproteins in OVCA432 cells. Antiproliferative	[118]
Thyroid Cancer	*Morus alba*		↑Autophagy-dependent cell death ↑Apoptosis	[119]
Prostate Cancer	*Vitis vinifera*	Proanthocyanidins	↓Notch1 pathway	[120]
	*Luempua*	C3G	↓Epithelial mesenchymal transition	[121]
Bladder Cancer	*Ipomoea batatas*		↓PI3K/Akt, Bcl-2 ↑Apoptosis	[122]
Non-small-cell lung cancer	*Vaccinium myrtillus*	Dp, Cy, Mv, Pe, Pt	↓NSCLC growth and the metastatic processes, targets mediating cell proliferation, invasion and apoptosis.	[123]

**Table 3:** Mechanisms of anthocyanins from different plants as antivirals and antimicrobials.

Diseases	Type of Bacteria or Virus	Plant Origin	Mechanism	Reference
Antimicrobial	*Pseudomonas aeruginosa* *Escherichia coli* *Proteus mirabilis* *Acinetobacter baumannii* *Staphylococcus aureus*	*Vaccinium corymbosum*	Interfering with microbial growth, hamper the adhesion to surfaces, with Staph	[131]
	*coli* *Morganella morganii* *aeruginosa* *E. faecalis* *E. faecium* *S. aureus*	*Maloideae subfamily*	Inhibited the development of biofilm	[132]
	*Escherichia coli* *Pseudomonas aeruginosa Staphylococcus aureus*	*Morus nigra*	Inhibitory effects on proinflammatory cytokines, iNOS and nuclear factor-κB (NF-κB) pathway-related proteins.	[133]
	*K. pneumoniae*	*Syzygium cumini*	Influencing the biofilm formation	[134]
	*Citrobacter freundii Enterococcus faecalis*	*Vaccinium myrtillus*, *Vaccinium corymbosum*		[135]
	*Staphylococcus aureus Pseudomonas aeruginosa* *Escherichia coli* *Candida albicans* *P. aeruginosa*	Black wheat Purple wheat Blue wheat	Inhibitory DNA replication, protein synthesis breaking cell wall	[136]
Antiviral	Viruses A (IVA) Viruses B (IVB)	Red-fleshed potato		[137]
	H1N1 subtypes of influenza virus H5N1-type influenza A virus SARS CoV-2	*Sambucus nigra*	Binding to H1N1 virions NA inhibition. Preventing reproduction	[138]
	Rabies virus	*Vigna angularis*	Affected early phase of infection cycle and viral infectivity	[139]
	HSV-1	*Rosaceae*	Extracellular mechanism	[140]

## Data Availability

Not available.

## Abbreviations

Cy	Cyanidin
C3G	Cyanidin-3-glucoside
C3R	Cyanidin-3-rutinoside
C-3-Ara	Cyanidin-3-arabinoside
C-3-Gal	Cyanidin-3-galactoside
C3X	Cyanidin-3-xylosyl
C3XR	Cyanidin-3-xylosylrutinoside
C-3-Sam	Cyanidin 3-sambubioside
C3GR	Cyanidin-3-glucosyl-rutinoside,
C3MG	Cyanidin-3-malonylglucoside
C3DG	Cyanidin-3-dioxalylglucoside
C-3-5-D	Cyanidin-3,5-diglucoside
Dp	Delphinidin
D3G	Delphinidin-3-glucoside
D3R	Delphinidin-3-rutinoside
D-3-Sam	Delphinidin 3-sambubioside
D-3-Gen	Delphinidin 3-gentiobioside
D-3-Neo	Delphinidin-3-neohesperidoside
D-3-Gal	Delphinidin-3-galactoside
D-3-Ara	Delphinidin-3-arabinoside
D-3-5-D	Delphinidin-3,5-diglucoside
Pn	Peonidin
Pn-3-G	Peonidin-3-glucoside
Pn-3-Gal	Peonidin 3-galactoside
Pn-3-Ara	Peonidin 3-arabinoside
Pn-3-5-D	Peonidin-3,5-diglucoside
Pt	Petunidin
Pt-3-G	Petunidin-3-glucosides
Pt-3-Gal	Petunidin-3-O-galactoside
Pt-3-5-D	Petunidin-3,5-diglucoside
Pt-3-RG	Petunidin-3-rutinoside-5-glucoside
Pg	Pelargonidin
Pg-3-G	Pelargonidin-3-glucoside
Pg-3-R	Pelargonidin-3-rutinoside
Pg-3-MG	Pelargonidin-3-malonylglucoside
Mv	Malvidin
Mv-3-G	Malvidin-3-glucoside
Mv-3-gal	Malvidin-3-galactoside
Mv-3-5-D	Malvdin-3,5-diglucoside
Mv-3-RG	Malvidin-3-rutinoside-5-glucoside
PA	Polymerized anthocyanin
